# Differential cell line susceptibility to the emerging Zika virus: implications for disease pathogenesis, non-vector-borne human transmission and animal reservoirs

**DOI:** 10.1038/emi.2016.99

**Published:** 2016-08-24

**Authors:** Jasper Fuk-Woo Chan, Cyril Chik-Yan Yip, Jessica Oi-Ling Tsang, Kah-Meng Tee, Jian-Piao Cai, Kenn Ka-Heng Chik, Zheng Zhu, Chris Chung-Sing Chan, Garnet Kwan-Yue Choi, Siddharth Sridhar, Anna Jinxia Zhang, Gang Lu, Kin Chiu, Amy Cheuk-Yin Lo, Sai-Wah Tsao, Kin-Hang Kok, Dong-Yan Jin, Kwok-Hung Chan, Kwok-Yung Yuen

**Affiliations:** 1 State Key Laboratory of Emerging Infectious Diseases, The University of Hong Kong, Hong Kong, China; 2 Department of Microbiology, The University of Hong Kong, Hong Kong, China; 3 Research Centre of Infection and Immunology, The University of Hong Kong, Hong Kong, China; 4 Carol Yu Centre for Infection, The University of Hong Kong, Hong Kong, China; 5 Department of Pathogen Biology, Hainan Medical University, Haikou, Hainan 571101, China; 6 Department of Ophthalmology, The University of Hong Kong, Hong Kong, China; 7 Resarch Centre of Heart, Brain, Hormone and Healthy Aging, The University of Hong Kong, Hong Kong, China; 8 State Key Laboratory of Brain and Cognitive Sciences, The University of Hong Kong, Hong Kong, China; 9 School of Biomedical Sciences, The University of Hong Kong, Hong Kong, China; 10 The Collaborative Innovation Center for Diagnosis and Treatment of Infectious Diseases, The University of Hong Kong, Hong Kong, China

**Keywords:** animal, cell line, flavivirus, placenta, transmission, tropism, virus, Zika

## Abstract

Zika virus (ZIKV) is unique among human-pathogenic flaviviruses by its association with congenital anomalies and trans-placental and sexual human-to-human transmission. Although the pathogenesis of ZIKV-associated neurological complications has been reported in recent studies, key questions on the pathogenesis of the other clinical manifestations, non-vector-borne transmission and potential animal reservoirs of ZIKV remain unanswered. We systematically characterized the differential cell line susceptibility of 18 human and 15 nonhuman cell lines to two ZIKV isolates (human and primate) and dengue virus type 2 (DENV-2). Productive ZIKV replication (⩾2 log increase in viral load, ZIKV nonstructural protein-1 (NS1) protein expression and cytopathic effects (CPE)) was found in the placental (JEG-3), neuronal (SF268), muscle (RD), retinal (ARPE19), pulmonary (Hep-2 and HFL), colonic (Caco-2),and hepatic (Huh-7) cell lines. These findings helped to explain the trans-placental transmission and other clinical manifestations of ZIKV. Notably, the prostatic (LNCaP), testicular (833KE) and renal (HEK) cell lines showed increased ZIKV load and/or NS1 protein expression without inducing CPE, suggesting their potential roles in sexual transmission with persistent viral replication at these anatomical sites. Comparatively, none of the placental and genital tract cell lines allowed efficient DENV-2 replication. Among the nonhuman cell lines, nonhuman primate (Vero and LLC-MK2), pig (PK-15), rabbit (RK-13), hamster (BHK21) and chicken (DF-1) cell lines supported productive ZIKV replication. These animal species may be important reservoirs and/or potential animal models for ZIKV. The findings in our study help to explain the viral shedding pattern, transmission and pathogenesis of the rapidly disseminating ZIKV, and are useful for optimizing laboratory diagnostics and studies on the pathogenesis and counter-measures of ZIKV.

## Introduction

Zika virus (ZIKV) is a mosquito-borne flavivirus that has been largely neglected for 60 years after its first isolation from the serum of a febrile sentinel rhesus macaque in the Zika Forest of Uganda in 1947.^1^ Since 2007, ZIKV has emerged to cause large epidemics of a dengue-like illness in the Pacific islands and Latin America, with imported cases also reported in other continents.^2, 3^ As of 14 July 2016, more than 60 countries/territories have reported continuing mosquito-borne transmission of ZIKV.^4^ Although most patients infected with ZIKV have an asymptomatic or a self-limiting acute febrile illness, some patients may develop severe neurological complications, such as Guillain–Barré syndrome, or fatal disseminated infections.^2, 5, 6, 7^ Moreover, recent clinico-epidemiological, laboratory and animal studies on pathogenesis have established the association between ZIKV infection and congenital anomalies, such as microcephaly, central nervous system malformations and ophthalmological abnormalities.^8, 9, 10, 11, 12, 13^

ZIKV research has so far been mainly focused on the pathogenesis of ZIKV-associated neurological complications. Questions on the pathogenesis of ZIKV's other clinical manifestations, its unique ability among flaviviruses to be transmitted trans-placentally and sexually, and its potential animal reservoirs remain mostly unanswered. Although *in vivo* phenomena may not be completely reproducible in the *in vitro* setting, cell line susceptibility studies are often useful for providing critical data for these knowledge gaps in the early phases of epidemics caused by emerging RNA viruses.^14, 15^ In this study, we correlated the differential cell line susceptibility, species tropism, viral replication efficiency and antigen expression patterns with the clinical and epidemiological characteristics of ZIKV, and compared them with those of the closely related dengue virus type 2 (DENV-2). Our findings provided novel implications on the disease pathogenesis, transmission and potential animal reservoirs that could be applied to optimize laboratory testing protocols and infection control strategies for this global health emergency.

## Materials and Methods

### VIRAL ISOLATES

To investigate on the differential cell line susceptibility to epidemic ZIKV and pre-epidemic ZIKV, we included a clinical isolate of ZIKV (Puerto Rico strain PRVABC59) obtained from a patient in the recent South American epidemic (ZIKV-PR; kindly provided by Brandy Russell and Barbara Johnson, Centers for Disease Control and Prevention, USA), and another pre-epidemic ZIKV strain isolated from a nonhuman primate in Uganda in 1947 (976 Uganda strain) (ZIKV-U; kindly provided by Tatjana Avšič Županc, University of Ljubljana, Slovenia, the European Virus Archive). An archived clinical isolate of DENV-2 was obtained from Department of Microbiology, The University of Hong Kong, for comparison with ZIKV, as the two viruses are closely related phylogenetically and have similar clinical manifestations. The ZIKV and DENV-2 isolates were amplified by three additional passages in Vero cells and C6/36 cells, respectively, to make working stocks of the viruses.

### ETHICS STATEMENT

Institutional Review Board approval for use of the vial isolates was obtained and all samples were anonymized. The cell lines used in this study were obtained from sources listed in Table 1.

### VIRAL CULTURE

Viral culture was performed as we previously described with modifications.^14^ Briefly, 33 cell lines derived from different tissues or organs and host species were each inoculated with one multiplicity of infection of the ZIKV and DENV-2 isolates for 1 h (Table 1). Non-attached virus was removed by washing the cells twice in phosphate-buffered saline (Gibco, Thermo Fisher Scientific, Waltham, MA, USA). The monolayer cells were maintained in minimum essential medium, Dulbecco's modified Eagle's medium (DMEM), DMEM/F12 or RPMI medium, with 1% fetal calf serum, depending on the type of cell line according to supplier's instructions (Gibco). The suspension cells were maintained in RPMI medium with 2% fetal calf serum (Gibco). All infected cell lines were incubated at 37 °C for five days, except C6/36 which was incubated at 28 °C. Cytopathic effects (CPE) were examined at one, three and five days post-virus inoculation (d.p.i) with inverted light microscopy.

### RNA EXTRACTION AND QUANTITATIVE REVERSE-TRANSCRIPTION PCR

Total nucleic acid (TNA) was extracted from culture supernatants of the 33 cell lines infected by ZIKV or DENV-2 at 1, 3 and 5 d.p.i. using EZ1 Virus Mini Kit v2.0 (Qiagen, Hilden, Germany) as we previously described.^16, 17^ Briefly, 200 μL of supernatant were used for extraction and the TNA was eluted in 60 μL of AVE buffer. Real-time one-step quantitative reverse-transcription polymerase chain reaction (qRT-PCR) was used for the detection of ZIKV and DENV-2 using QuantiNova Probe RT-PCR Kit (Qiagen) in a LightCycler 96 Real-Time PCR System (Roche Diagnostics, Basel, Switzerland). Five microliters of purified TNA was amplified in a 20 μL-reaction containing 10 μL of 2 × QuantiNova Probe RT-PCR Master Mix, 0.2 μL QN Probe RT-mix, 0.8 μM forward primer, 0.8 μM reverse primer and 200 nM probe. ZIKV detection was performed as previously described, with slight modifications.^18^ Forward primer (5′-CGY TGC CCA ACA CAA GG-3′), reverse primer (5′-CCA CYA AYG TTC TTTT GCA BAC A-3′) and probe (5′-HEX-AGC CTA CCT TGA YAA GCA RTC AGA CAC TC-IABkFQ-3′) targeting the ZIKV envelope gene were used. For DENV-2 detection, forward primer (5′-GCA TAT TGA CGC TGG GAR AGA C-3′), reverse primer (5′-CGY TCT GTG CCT GGA WWG ATG-3′) and probe (5′-FAM-CAG AGA TCC TGC TGT C-MGB-NFQ-3′) targeting the DENV-2 3'-untranslated region were used. Reactions were incubated at 45 °C for 10 min, followed by 95 °C for 5 min, and then thermal cycled for 50 cycles (95 °C for 5 s, 55 °C for 30 s). A series of 10-fold dilutions equivalent to 1 × 10^2^–1 × 10^6^ copies/reaction mixture were prepared to generate calibration curves and run in parallel with the test samples.

### CLONING AND PURIFICATION OF HIS6-TAGGED RECOMBINANT ZIKV AND DENV-2 NS1

Primers (5′-CAT ATG GAT GTG GGG TGC TCG GTG GAC T-3′ and 5′-CTC GAG TGC AGT CAC CAT TGA CCT TAC T-3′) were used to amplify the gene encoding the ZIKV–nonstructural protein 1 (NS1) protein by RT-PCR. The sequence encoding a 352-amino-acid fragment of ZIKV–NS1 protein was amplified and cloned into the *Nde*I and *Xol*I sites of expression vector pETH in frame and downstream of the series of six histidine residues. The recombinant ZIKV–NS1 protein was expressed in *Escherichia coli*, denatured in 8 M urea, and purified with Ni-nitrilotriacetic acid affinity chromatography (Qiagen) according to the manufacturer's instructions. The solubilized protein was refolded by slowly dialyzing with refolding buffers. Expression of the recombinant ZIKV–NS1 protein was confirmed by western blot analysis using mouse anti-His monoclonal antibody (Sigma-Aldrich, St Louis, MO, USA). Preparation of DENV-2-NS1 protein was performed as previously described.^19^

### PREPARATION OF SPECIFIC ANTIBODIES AGAINST ZIKV- AND DENV-2-NS1 PROTEINS

This was performed as we previously described with modifications.^14^ Briefly, 30 μg of purified recombinant ZIKV-NS1 protein was mixed with an equal volume of complete Freund's adjuvant (Sigma-Aldrich) and injected subcutaneously into BALB/c mice. Incomplete Freund's adjuvant (Sigma-Aldrich) was used in subsequent injections at 14-day intervals for three times. Serum samples were collected 14 days after the fourth injection. Preparation of specific antibodies against DENV-2-NS1 protein was performed as previously described.^19^

### ANTIGEN DETECTION OF INFECTED CELL LINES BY IMMUNOFLUORESCENCE

This was performed as we previously described with modifications.^14^ Cell smears at 1, 3 and 5 d.p.i. were prepared and fixed in chilled acetone at −20 °C for 10 min. The fixed cells were incubated with mouse antiserum against the ZIKV- or DENV-2-NS1 protein, followed by FITC-rabbit anti-mouse IgG (Invitrogen, Carlsbad, CA, USA). Cells were then examined under a fluorescence microscope. Three representative microscopic fields were chosen and 100 cells were counted for characteristic cytoplasmic apple green fluorescence. The mean percentage of positive cells was rounded up to the nearest multiplicity of 10. Uninoculated cells were used as negative control.

### STATISTICAL ANALYSIS

The Student's *t*-test was used to compare the mean viral load of the different cell lines at 1, 3 and 5 d.p.i. with the mean baseline viral load at 0 d.p.i. All calculations were based on log-transformed viral loads. *P-*value of <0.01 was considered as statistically significant. Computation was performed using the Predictive Analytics Software v18.0 for Windows.

## Results

### HUMAN CELL LINES

**Figure 1:**
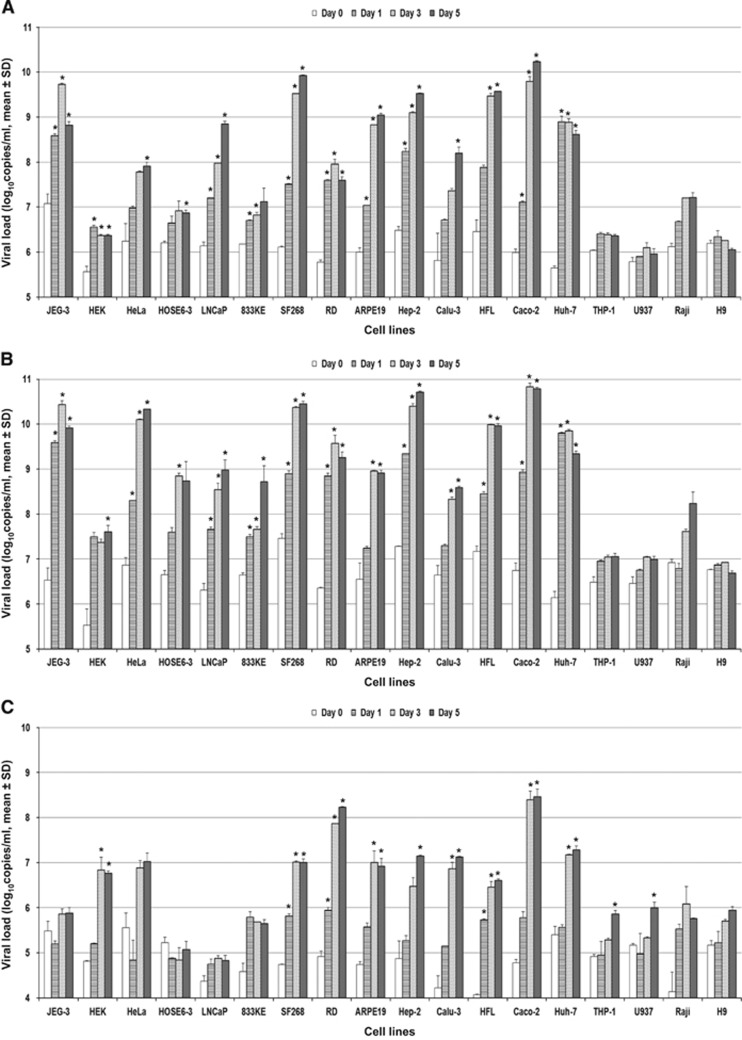
Differential human cell line susceptibility to (**A**) ZIKV-PR, (**B**) ZIKV-U and (**C**) DENV-2, as defined by viral load, on days 1, 3 and 5 post-ZIKV inoculation. All experiments were done in triplicate. The mean viral loads on days 1, 3 and 5 were compared with the mean baseline viral load on day 0 (1 h post-ZIKV inoculation). All calculations were based on log-transformed viral loads. *P-value of <0.01.

**Figure 2:**
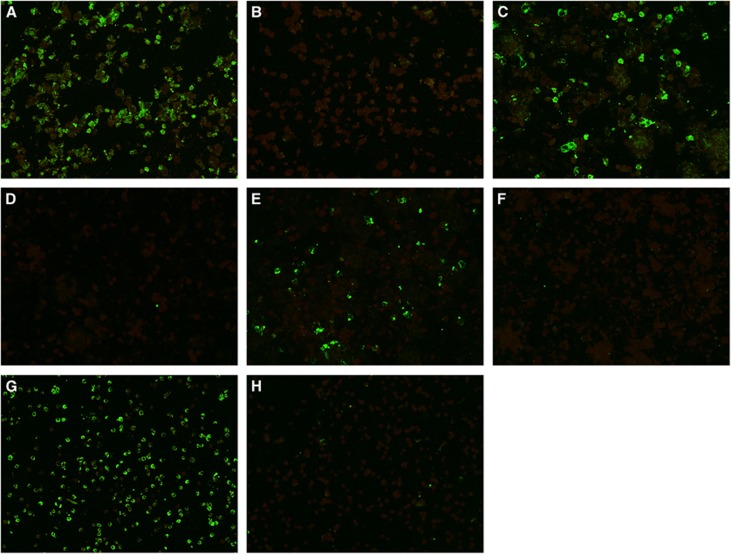
ZIKV nonstructural protein 1 expression by immunofluorescence in cell lines representing the potential trans-placental, sexual and vector-borne transmission routes, and/or genitourinary tissues with persistent viral replication of ZIKV. Expression of ZIKV nonstructural protein 1 as intense apple green cytoplasmic fluorescence in different cell lines stained by monospecific polyclonal serum from BALB/c mice immunized with His6-tagged recombinant ZIKV nonstructural protein 1 at day 5 post-ZIKV inoculation (original magnification × 200). (**A**) Infected placental (JEG-3) cells; (**B**) uninfected JEG-3 control; (**C**) infected prostatic (LNCaP) cells; (**D**) uninfected LNCaP control; (**E**) infected renal (HEK) cells; (**F**) uninfected HEK control; (**G**) infected mosquito (C6/36) cells; (**H**) uninfected C6/36 control.

**Figure 3:**
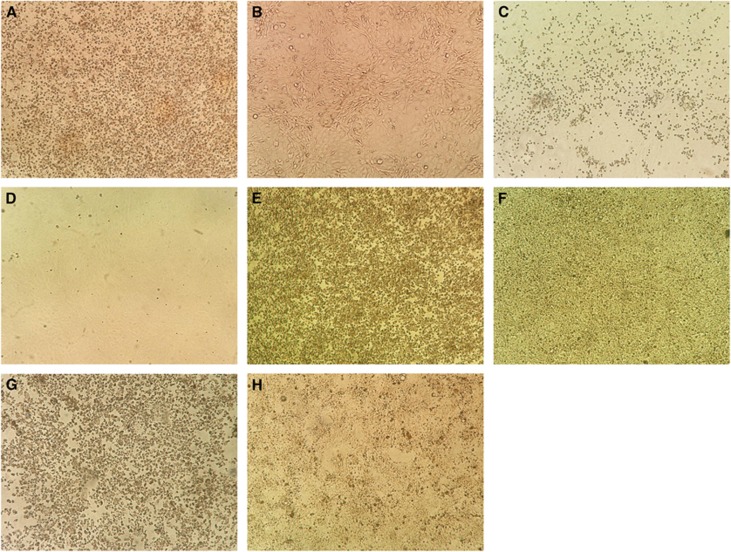
Cytopathic effects in cell lines representing the major organs with clinical manifestations in ZIKV infection. Cytopathic effects consisting of cell rounding, detachment and degeneration were observed in different cell lines at day 5 post-ZIKV inoculation under inverted microscopy (original magnification × 100). (**A**) Infected neuronal (SF268) cells; (**B**) uninfected SF268 control; (**C**) infected retinal (ARPE19) cells; (**D**) uninfected ARPE19 control; (**E**) infected muscle (RD) cells; (**F**) uninfected RD3 control; (**G**) infected hepatic (Huh-7) cells; (**H**) uninfected Huh-7 control.

Eighteen human cell lines were tested (Table 1). ZIKV-PR and ZIKV-U showed similar tropism as defined by viral load, immunofluorescent antigen staining and CPE. A ⩾2 log increase in mean viral load (*P*<0.01) within 5 d.p.i. was observed in 14/18 cell lines infected with ZIKV-PR or ZIKV-U (Figures 1A and 1B). These included the placental (JEG-3), genitourinary (HEK, HeLa, HOSE6-3, LNCaP and 833KE), neuromuscular (SF268 and RD), retinal (ARPE19), respiratory (Hep2, Calu-3 and HFL), intestinal (Caco-2) and hepatic (Huh-7) cell lines. There was a slight increase in mean viral load of about 0.5–1 lgcopies/mL in the monocyte (THP-1) and B lymphocyte (Raji) cell lines (*P*>0.01), but none of the immune cell lines achieved a ⩾2 log increase in mean viral load within 5 d.p.i. The highest mean viral loads (⩾10 lgcopies/mL) were observed in the placental (JEG-3), cervical (HeLa), neuronal (SF268), upper respiratory tract (Hep-2) and intestinal (Caco-2) cell lines. Twelve of these 14 cell lines (JEG-3, HEK, HeLa, LNCaP, SF268, RD, ARPE19, Hep-2, Calu-3, HFL, Caco-2 and Huh-7) showed obvious ZIKV-NS1 protein expression by IF in addition to a high viral load (Table 2). The peak ZIKV-NS1 protein expression in these cell lines occurred on 3–5 d.p.i. ZIKV-NS1 protein expression was most prominent (⩾50% of infected cells) in the placental (JEG-3), neuronal (SF268), muscle (RD), retinal (APRE19), lower respiratory tract (HFL) and hepatic (Huh-7) cell lines (Figure 2). Although ovarian (HOSE6-3) and testicular (833KE) cells showed high mean viral loads in qRT-PCR, ZIKV-NS1 protein expression was consistently found in only ⩽5% of infected cells. CPE were observed in 8 of these 14 cell lines, namely, JEG-3, SF268, RD, ARPE19, Hep-2, HFL, Caco-2 and Huh-7 (Table 3). CPE were most prominent (>50% involvement) in the placental (JEG-3), neuronal (SF268), muscle (RD), intestinal (Caco-2) and hepatic (Huh-7) cell lines (Figure 3). The gradual onset of CPE in most of these cell lines at 3 d.p.i. correlated with the steady increase of viral load and ZIKV-NS1 protein expression at 3–5 d.p.i.

In contrast, DENV-2 showed a much narrower range of human tissue tropism, with ⩾2 log increase in the mean viral load (*P*<0.01) in only 9/18 cell lines (HEK, SF268, RD, ARPE19, Hep-2, Calu-3, HFL, Caco-2 and Huh-7; Figure 1C). Notably, unlike ZIKV, the DENV-2 load did not significantly increase by ⩾2 lgcopies/mL in any of the genital tract and placental cell lines, with only mildly increased viral load (*P*>0.01) and DENV-2-NS1 protein expression in the HeLa cell line. Most of these nine cell lines with increased viral loads demonstrated DENV-2-NS1 protein expression (Table 2) and CPE (Table 3). The CPE were generally delayed in onset (5 d.p.i.) and limited (⩽50%), except in the RD and Huh-7 cell lines.

### NONHUMAN CELL LINES

**Figure 4:**
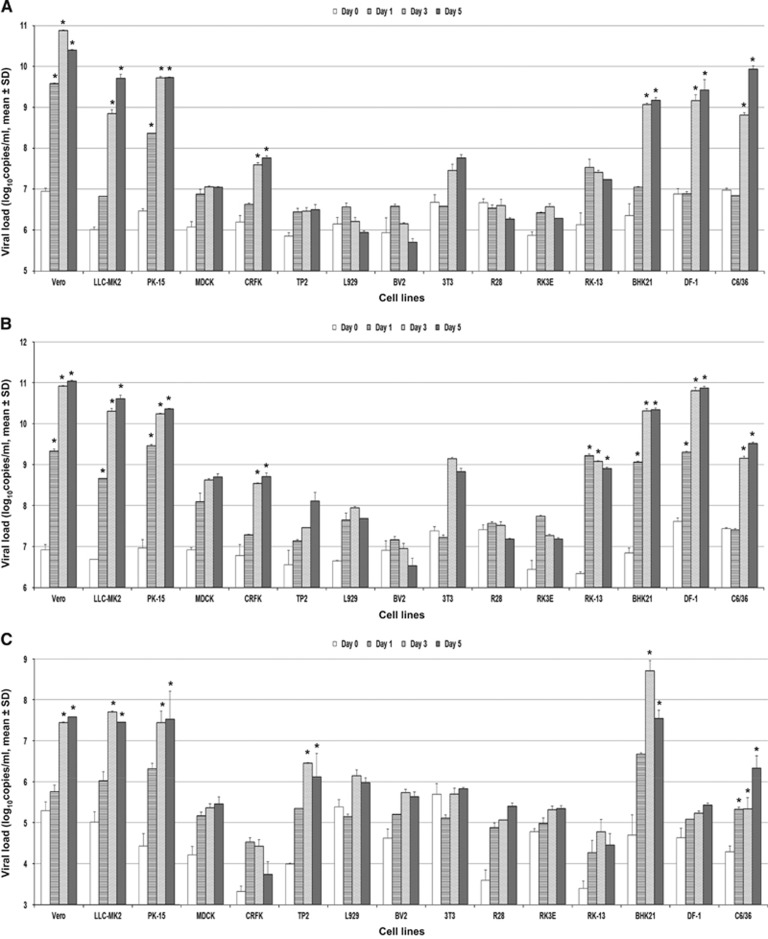
Differential nonhuman cell line susceptibility to (**A**) ZIKV-PR, (**B**) ZIKV-U and (**C**) DENV-2, as defined by viral load, on days 1, 3 and 5 post-ZIKV inoculation. All experiments were done in triplicate. The mean viral loads on days 1, 3 and 5 were compared with the mean baseline viral load on day 0 (1 h post-ZIKV inoculation). All calculations were based on log-transformed viral loads. *P-value of <0.01.

Fifteen nonhuman cell lines were tested (Table 1). ZIKV-PR and ZIKV-U again showed similar tropism in these cell lines. A ⩾2 log increase in mean viral load (*P*<0.01) was observed within 5 d.p.i. in 8/15 cell lines (Figures 4A and 4B). These included the nonhuman primate (Vero and LLC-MK2), pig (PK-15), cat (CRFK), rabbit (RK-13), hamster (BHK21), chicken (DF-1) and mosquito (C6/36) cell lines. None of the mouse, rat and bat cell lines showed a ⩾2 log increase in viral load within 5 d.p.i. The highest mean viral loads (⩾10 lgcopies/mL) were observed in the nonhuman primate (Vero and LLC-MK2), pig (PK-15), hamster (BHK21) and chicken (DF-1) cell lines. All of these eight cell lines showed ZIKV-NS1 protein expression by IF, usually starting on 3 d.p.i. and peaked on 5 d.p.i. (Table 2). ZIKV-NS1 protein expression was most prominent (⩾50% of infected cells) in the nonhuman primate (Vero and LLC-MK2), pig (PK-15), hamster (BHK21), chicken (DF-1) and mosquito (C6/36) cell lines (Figure 2). Some of the mouse, rat and bat cell lines also showed ZIKV-NS1 protein expression, but these expressions were much less prominent than that in the other cell lines. CPE were observed in six of these eight cell lines, including the nonhuman primate (Vero and LLC-MK2), pig (PK-15), rabbit (RK-13), hamster (BHK21) and chicken (DF-1) cell lines, and were most prominent (>50% involvement) in the nonhuman primate, pig, hamster and chicken cell lines (Table 3). CPE were not observed in the mosquito (C6/36) cell line within 5 d.p.i.

A narrower range of nonhuman cell lines were susceptible to infection by DENV-2 than ZIKV. A ⩾2 log increase in mean viral load (*P*<0.01) within 5 d.p.i. was observed in only 6/15 cell lines, including the nonhuman primate (Vero and LLC-MK2), pig (PK-15), bat (TP2), hamster (BHK21) and mosquito (C6/36) cell lines (Figure 4C). All of these six cell lines demonstrated DENV-2-NS1 protein expression by IF (Table 2). CPE were observed in five of these six cell lines (all except C6/36), but was usually not prominent (⩽50%) except in the LLC-MK2 and BHK21 cell lines (Table 3).

## Discussion

Although ZIKV infection has been described as a dengue-like illness in the past, recent evidence suggests that ZIKV is unique among known human-pathogenic flaviviruses. ZIKV is the first flavivirus known to be associated with congenital malformations, and sexual and vertical transmissions in human.^1, 8, 9, 20, 21, 22^ The pathogenesis of these unusual clinical manifestations and transmission routes is incompletely understood. Moreover, a comprehensive surveillance for potential animal reservoirs of ZIKV, which are likely important in facilitating the spread of the virus after 60 years of quiescence, has not been reported. A virus' ability to grow in cells of different host species might provide insight into its ability to cross interspecies barriers. We therefore conducted this systematic study to characterize the tropism of the emerging ZIKV in a broad range of human and nonhuman cell lines derived from different tissues or organs and host species. The findings in our study may have important clinical and epidemiological implications for this rapidly expanding viral epidemic.

Productive ZIKV replication as defined by ⩾2 log increase in mean viral load, ZIKV-NS1 protein expression by IF, and presence of CPE was observed within 5 d.p.i. in 8/18 human cell lines, which represented cells of placental, neuronal, muscle, retinal, pulmonary, intestinal and hepatic origin. This broad tissue tropism is likely related to the wide distribution of the candidate receptors of ZIKV, including AXL, TYRO3 and DC-SIGN, in different organs.^23^ The placental (JEG-3) and neuronal (SF268) cell line tropism corroborated with the congenital and neurological manifestations of ZIKV infection. These manifestations include congenital microcephaly and other central nervous system malformations in infected fetuses, and meningoencephalitis, myelitis, and post-infectious Guillain–Barré syndrome in adults.^1, 5, 8, 24^ ZIKV RNA, full-length viral genome and/or viral particles could be detected in the brain tissue of fetuses with congenital microcephaly.^8, 25^ Microscopically, flavivirus-like virions were observed together with extensive inflammation, astrocyte and microglial activation, and injury in the cortex and the lateral corticospinal tracts in the fetal brain tissue.^8, 25^ Moreover, ZIKV could impair growth and deplete neural progenitor cells in human neurospheres and cerebral organoids.^26, 27^

Consistent with ZIKV's tropism for the human rhabdomyosarcoma (RD) cell line, myalgia and arthralgia with periarticular edema are common features in symptomatic ZIKV infection. Fetuses with congenital ZIKV infection may develop arthrogryposis.^28^ Myositis and myocarditis were observed in suckling mice inoculated with ZIKV intracerebrally, and viral RNA could be detected in the muscle and heart of type I interferon receptor-deficient mice infected with ZIKV.^29, 30^

Viral tropism for the retinal (ARPE19) cell line corroborates with the ophthalmological complications of congenital ZIKV infection, including chorioretinal atrophy and pigmentary maculopathy.^10, 13^ Abdominal symptoms including diarrhea, vomiting, abdominal pain and jaundice with hepatic dysfunction have been reported in patients with ZIKV infection, and are in agreement with the susceptibility of the intestinal (Caco-2) and hepatic (Huh-7) cell lines to ZIKV.^1, 6, 7, 31^ Respiratory distress may occur in severe ZIKV infection, and this correlates with ZIKV's tropism for respiratory (Hep-2, Calu-3 and HFL) cell lines.^6, 7^ Similarly, the clinical features of DENV infection, including encephalopathy/encephalitis (SF268), myalgia and rhabdomyolysis (RD), retinitis (ARPE19), respiratory symptoms (Hep-2 and HFL), diarrhea (Caco-2) and hepatic dysfunction (Huh-7), also correlated with the cell line susceptibility to DENV-2 in our study.^32, 33^

Although neurological manifestations may also be seen in infections caused by other flaviviruses, such as DENV, yellow fever virus, West Nile virus (WNV) and Japanese encephalitis virus (JEV), none of these flaviviruses have been associated with congenital microcephaly and central nervous system malformations. These unique clinical features of ZIKV infection require the virus to be able to infect neural progenitor cells of the fetus in utero during early gestation. A striking difference between the cell line susceptibilities of ZIKV and DENV-2 observed in our study was ZIKV's ability to productively replicate in placental cells (JEG-3). JEG-3 cells infected with ZIKV produced high viral loads (~10 lgcopies/mL), abundant ZIKV-NS1 protein expression and prominent CPE. This finding helped to explain the unique ability of ZIKV to infect the fetus via the placenta and cause congenital anomalies, particularly when the infection occurred during the first trimester.^34^ Although a previous study suggested that type III interferons produced by human placental trophoblasts could confer protection against ZIKV infection, it is notable that the study utilized primary human trophoblasts of full-term instead of early gestation placenta.^35^ Indeed, our *in vitro* finding concurred with the recent reports of detectable viral particles and RNA in trophoblasts of the maternal and fetal placenta of ZIKV-infected type I interferon signaling-deficient mice and permissibility of placental macrophages and cytotrophoblasts to productive ZIKV infection.^12, 36^

Besides trans-placental transmission, our findings also provided insights to explain the pathogenesis of other non-vector-borne human transmission routes of ZIKV. Probable sexual transmission of ZIKV has been reported.^20, 21, 22^ ZIKV-infected male patients might develop hematospermia and perineal pain compatible with prostatitis, and might have detectable viral RNA and/or infectious viral particles in their semen.^20, 21, 22^ In contrast to DENV-2, which did not efficiently replicate in any of the genital tract cell lines, the mean ZIKV load significantly increased in the prostatic (LNCaP) and testicular (833KE) cell lines without inducing CPE. This suggested that the prostate and testes might be important sites of ZIKV replication that led to prolonged virus shedding in the semen of infected men.^20, 21, 37^ Interestingly, abundant ZIKV-NS1 protein expression was detected in ZIKV-infected LNCaP cells, but not 833KE cells. This finding might imply that ZIKV could replicate more efficiently in the prostate than the testes. Recent studies only reported high viral loads in the testes of ZIKV-infected mice deficient in interferon signaling and/or receptor, but did not report the viral loads in their prostates.^30, 38^ Further studies should be conducted to investigate the prostate's role as a replication site of ZIKV. The female genital tract cell lines, including HeLa and HOSE6-3, were also susceptible to ZIKV, supporting the hypothesis that non-immune women might acquire the infection through sexual intercourse with their ZIKV-infected male partners.

We showed that the renal (HEK) and respiratory tract (Hep-2, Calu-3, HFL) cell lines were also susceptible to ZIKV. This helped to explain the high viral loads in the urine and nasopharyngeal swabs of ZIKV-infected patients.^39, 40, 41^ The finding of high viral load and abundant ZIKV-NS1 protein expression without inducing CPE in HEK cells is particularly interesting, as it suggests that the kidney might be another site of persistent ZIKV replication. Similarly, DENV-2 infection also caused increased mean viral load and DENV-2-NS1 protein expression without inducing CPE in HEK cells, and viral RNA might be detected in urine of dengue patients.^42^ An important implication of this finding is that kidney transplant donors in ZIKV-affected regions should be tested for the infection before organ donation, as virus shedding in urine might be prolonged.^39, 40, 41^ The intestinal (Caco-2) cell line was also highly susceptible to ZIKV, which corroborated with the high viral load detected in the intestines of ZIKV-infected mice.^43^ Serial testing of fecal samples of ZIKV-infected patients should be performed to determine whether feces represent another possible transmission route of ZIKV. It would also be interesting to investigate ZIKV's ability to productively replicate in salivary gland cells, as high viral load could also be detected in the saliva of ZIKV-infected patients.^41, 44^

The differential cell line susceptibility of our nonhuman cell lines to ZIKV may provide insights into the virus' potential animal reservoirs and alternative animal models. The finding of high viral loads and abundant ZIKV-NS1 protein expression without induction of CPE within 5 d.p.i. in the *Aedes albopictus* (C6/36) cell line was quite expected, and corroborated with the high non-fatal infection, dissemination and transmission rates of ZIKV in infected *A. albopictus*.^45^ A ⩾2 log increase in mean viral load with ZIKV-NS1 protein expression and CPE were observed in the nonhuman primate (Vero and LLC-MK2), pig (PK-15), rabbit (RK-13), hamster (BHK21) and chicken (DF-1) cell lines. It is well known that the transmission of flaviviruses may involve sylvatic cycles between the mosquito vectors and susceptible wild animals, and urban cycles between the vectors and domestic animals.^46, 47^ Nonhuman primates are known wild animal reservoirs of ZIKV and DENV.^1, 46^ Our findings of efficient ZIKV replication in pig, rabbit, hamster and chicken cell lines suggest that these mammalian or avian species might have a role in the transmission of ZIKV. Similar to domestic pigs and wild birds for JEV, and domestic and wild birds for WNV, these species may serve as the animal reservoirs or amplifying hosts of ZIKV.^46^ Moreover, vector-free JEV transmission due to persistence of virus in tonsils and virus shedding in oronasal secretions of pigs has recently been reported.^48^ Alternatively, these animal species may be susceptible hosts with less importance in the transmission cycle, which is analogous to horses for JEV and many vertebrate species for WNV.^46^ Systematic sampling of domestic animals in ZIKV-affected regions would help to confirm these findings. Pigs, rabbits and hamsters should be evaluated as animal models for ZIKV infection, as interferon signaling- or receptor-deficient mice are not routinely available in most laboratories. Pig and/or hamster models have been previously reported for DENV and WNV infection.^49, 50^

Our findings are useful for optimizing laboratory diagnostics and studies on the pathogenesis and counter-measures of ZIKV. Some of the laboratories in resource-limited areas affected by ZIKV may not have the capability to routinely perform molecular diagnostic tests. In addition to the few cell lines previously reported, our study has identified a broad range of cell lines that are suitable for growing ZIKV as an alternative diagnostic tool in clinical virology laboratories.^51, 52, 53, 54^ These cell lines may also be used in pathogenesis studies conducted by laboratories without the technical expertise to handle brain organoids, and to evaluate antiviral treatment and vaccines *in vitro* prior to animal experiments.

## Figures and Tables

**Table 1:** Human and nonhuman cell lines used in the present study

**Organism, anatomic site, cell line**	**Abbreviations**	**Source**
*Human*		
Placenta		
Placental choriocarcinoma	JEG-3	ATCC no. HTB-36
Genitourinary tract		
Fetal kidney	HEK	In-house
Cervical adenocarcinoma	HeLa	ATCC no. CCL-2.2
Ovarian surface epithelium	HOSE6-3	In-house
Metastatic prostatic adenocarcinoma	LNCaP	ATCC no. CRL-1740
Testicular germ cell tumor	833KE	Sigma-Aldrich, St Louis, MO, USA
Neuromuscular cells		
Anaplastic astrocytoma (non-epithelial)	SF268	Research Center for Emerging Viral Infections, Chang Gung University, Taiwan
Rhabdomyosarcoma	RD	ATCC no. CCL-136
Retina		
Retinal pigment epithelium	ARPE19	ATCC no. CRL-2302
Respiratory tract		
Laryngeal epidermoid carcinoma	Hep-2	ATCC no. CCL-23
Lung adenocarcinoma	Calu-3	ATCC no. HTB-55
Embryonic lung fibroblasts	HFL	In-house
Gastrointestinal tract		
Colorectal adenocarcinoma	Caco-2	ATCC no. HTB-37
Liver		
Hepatocellular carcinoma	Huh-7	JCRB cell bank of Okayama University, Japan
Immune cells		
Peripheral blood monocytes from acute monocytic leukemia	THP-1	ATCC no. TIB-202
Monocytes from histiocytic lymphoma	U937	ATCC no. CRL-1593.2
B lymphocytes from Burkitt's lymphoma	Raji	ATCC no. CCL-86
T lymphocytes	H9	ATCC no. HTB-176
		
*Nonhuman*		
Mammals		
African green monkey kidney	Vero	ATCC no. CCL-81
Rhesus monkey kidney	LLC-MK2	ATCC no. CCL-7
Porcine kidney	PK-15	ATCC no. CCL-33
Madin–Darby canine kidney	MDCK	ATCC no. CCL-34
Crandell Rees feline kidney	CRFK	ATCC no. CCL-94
Bat brain	TP2	In-house
Mouse subcutaneous areolar and adipose tissue	L929	ATCC no. CCL-1
Immortalized mouse microglia	BV2	Cell Resource Center, Institute of Basic Medical Sciences, Chinese Academy of Medical Sciences/Peking Union Medical College
Primary mouse embryonic fibroblasts	3T3	ATCC no. CCL-92
Rat retinal precursor cells	R28	Kerafast, Inc., Boston, MA, USA
Rat kidney	RK3E	ATCC no. CRL-1895
Rabbit kidney	RK-13	ATCC no. CCL-37
Baby hamster kidney	BHK21	ATCC no. CCL-10
Others		
Chicken fibroblasts	DF-1	ATCC no. CRL-12203
Mosquito (*Aedes albopictus*)	C6/36	ATCC no. CRL-1660

Abbreviation: American Tissue Culture Collection, ATCC.

**Table 2:** Differential cell line susceptibility to ZIKV-PR, ZIKV-U and DENV-2, as defined by immunofluorescent antigen staining on days 1, 3 and 5 after infection

**Cell lines**	**Immunofluorescence**^a^								
	**ZIKV-PR**			**ZIKV-U**			**DENV-2**		
	**Day 1**	**Day 3**	**Day 5**	**Day 1**	**Day 3**	**Day 5**	**Day 1**	**Day 3**	**Day 5**
*Human*									
Placenta									
JEG-3 (placenta)	50	60	80	60	70	80	N	N	N
Genitourinary tract									
HEK (kidney)	<1	1	5	10	20	20	1	20	20
HeLa (cervix)	N	<1	1	5	20	30	10	30	30
HOSE6-3 (endometrium)	1	1	1	1	5	5	N	N	N
LNCaP (prostate)	1	5	40	1	20	30	N	N	N
833KE (testis)	N	1	1	N	N	5	N	N	N
Neuromuscular cells									
SF268 (neuron)	5	20	40	30	70	80	40	40	30
RD (muscle)	1	10	20	30	70	50	50	90	80
Retina									
ARPE19 (retina)	5	60	50	20	60	60	10	30	30
Respiratory tract									
Hep-2 (larynx)	1	5	10	10	30	30	10	20	50
Calu-3 (lung)	1	5	10	1	5	10	10	20	10
HFL (lung)	5	40	60	10	60	60	40	40	40
Gastrointestinal tract									
Caco-2 (colon)	10	40	40	30	40	30	5	50	70
Liver									
Huh-7 (liver)	20	60	100	50	80	100	50	80	80
Immune cells									
THP-1 (monocyte)	<1	<1	<1	N	1	1	N	N	N
U937 (monocyte)	N	N	N	N	N	N	N	N	N
Raji (B lymphocyte)	N	N	<1	<1	<1	5	N	1	40
H9 (T lymphocyte)	N	N	N	N	<1	N	N	N	<1

*Nonhuman*									
Mammals									
Vero (monkey)	40	50	70	70	80	90	50	90	100
LLC-MK2 (monkey)	1	20	30	20	90	90	40	80	70
PK-15 (pig)	10	40	40	30	70	70	20	60	70
MDCK (dog)	1	1	5	5	10	10	N	N	5
CRFK (cat)	N	1	1	N	5	5	N	N	5
TP2 (bat)	N	N	N	N	5	10	20	30	30
L929 (mouse)	N	N	N	5	5	1	1	1	1
BV2 (mouse)	N	N	N	N	N	N	5	5	N
3T3 (mouse)	1	5	10	5	20	10	<1	<1	<1
R28 (rat)	N	N	N	N	N	N	<1	<1	<1
RK3E (rat)	5	5	5	10	10	10	N	<1	<1
RK-13 (rabbit)	20	30	20	40	40	30	N	N	N
BHK21 (hamster)	10	70	80	30	50	50	50	50	50
Others									
DF-1 (chicken)	1	30	70	40	70	70	N	<1	<1
C6/36 (mosquito)	10	40	90	10	60	90	N	30	40

a N is defined as negative. The numerals denote the mean percentage of positive cells from three representative microscopic fields, rounded up to the nearest multiplicity of 10.

**Table 3:** Differential cell line susceptibility to ZIKV-PR, ZIKV-U and DENV-2, as defined by cytopathic effect (CPE) on days 1, 3 and 5 after infection

**Cell lines**	**CPE, grade**^a^								
	**ZIKV-PR**			**ZIKV-U**			**DENV-2**		
	**Day 1**	**Day 3**	**Day 5**	**Day 1**	**Day 3**	**Day 5**	**Day 1**	**Day 3**	**Day 5**
*Human*									
Placenta									
JEG-3 (placenta)	N	1+	4+	1+	4+	4+	N	N	N
Genitourinary tract									
HEK (kidney)	N	N	N	N	N	N	N	N	N
HeLa (cervix)	N	N	N	N	1+	1+	N	N	N
HOSE6-3 (endometrium)	N	N	N	N	N	N	N	N	N
LNCaP (prostate)	N	N	N	N	N	N	N	N	N
833KE (testis)	N	N	N	N	N	N	N	N	N
Neuromuscular cells									
SF268 (neuron)	N	2+	2+	N	2+	3+	N	1+	2+
RD (muscle)	N	1+	2+	N	2+	4+	N	4+	4+
Retina									
ARPE19 (retina)	N	1+	1+	N	1+	2+	N	1+	2+
Respiratory tract									
Hep-2 (larynx)	N	N	N	N	1+	1+	N	N	1+
Calu-3 (lung)	N	N	N	N	N	N	N	N	1+
HFL (lung)	N	1+	1+	N	2+	3+	N	2+	2+
Gastrointestinal tract									
Caco-2 (colon)	N	N	3+	N	3+	4+	N	N	1+
Liver									
Huh-7 (liver)	N	3+	4+	1+	4+	4+	N	2+	4+
Immune cells									
THP-1 (monocyte)	N	N	N	N	N	N	N	N	N
U937 (monocyte)	N	N	N	N	N	N	N	N	N
Raji (B lymphocyte)	N	N	N	N	N	N	N	2+	4+
H9 (T lymphocyte)	N	N	N	N	N	N	N	N	1+

*Nonhuman*									
Mammals									
Vero (monkey)	N	3+	4+	N	4+	4+	N	1+	1+
LLC-MK2 (monkey)	N	1+	2+	N	2+	4+	N	4+	4+
PK-15 (pig)	N	2+	2+	N	2+	3+	N	1+	2+
MDCK (dog)	N	N	1+	N	1+	1+	N	N	1+
CRFK (cat)	N	N	N	N	N	N	N	1+	1+
TP2 (bat)	N	N	N	N	N	N	N	1+	2+
L929 (mouse)	N	N	N	N	N	N	N	N	N
BV2 (mouse)	N	N	N	N	N	N	N	N	N
3T3 (mouse)	N	N	N	N	N	N	N	N	N
R28 (rat)	N	N	N	N	N	N	N	1+	1+
RK3E (rat)	N	N	N	N	N	N	N	N	N
RK-13 (rabbit)	N	N	1+	N	1+	1+	N	N	N
BHK21 (hamster)	N	1+	2+	N	4+	4+	1+	4+	4+
Others									
DF-1 (chicken)	N	N	1+	N	4+	4+	N	N	N
C6/36 (mosquito)	N	N	N	N	N	N	N	N	N

a N is defined as negative, 1+ is defined as 1–25% involvement, 2+ is defined as >25% to 50% involvement, 3+ is defined as >50% to 75% involvement and 4+ is defined as >75% involvement.
